# 2-[(4-Methoxy­phen­yl)imino­meth­yl]-4-nitro­phenol

**DOI:** 10.1107/S1600536809008150

**Published:** 2009-03-11

**Authors:** Işın Kılıç, Erbil Ağar, Ferda Erşahin, Şamil Işık

**Affiliations:** a Department of Physics, Faculty of Arts and Sciences, Ondokuz Mayıs University, TR-55139 Kurupelit-Samsun, Turkey; b Department of Chemistry, Faculty of Arts and Sciences, Ondokuz Mayıs University, 55139 Samsun, Turkey

## Abstract

The title Schiff base compound, C_14_H_12_N_2_O_4_, is in an inter­mediate state between NH and OH tautomers. Apart from the intra­molecular O—H⋯N hydrogen bond, there are inter­molecular C—H⋯O hydrogen bonds, generating centrosymmetric *R*_2_^2^(18) and *R*_2_^2^(14) dimers.

## Related literature

For a related structure, see: Karabıyık *et al.* (2007 ▶). For geometric parameters, see: Allen *et al.* (1987 ▶); Glidewell *et al.* (2004 ▶); Zeller & Hunter (2004 ▶).
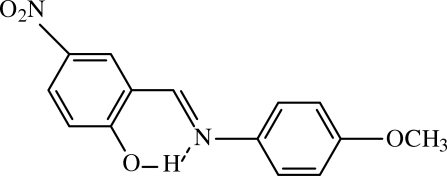


## Experimental

### 

#### Crystal data

C_14_H_12_N_2_O_4_
*M* *_r_* = 272.26
Monoclinic, 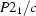

*a* = 3.8883 (3) Å
*b* = 21.6202 (17) Å
*c* = 15.3127 (11) Å
β = 97.887 (1)°
*V* = 1275.10 (17) Å^3^
*Z* = 4
Mo *K*α radiation
μ = 0.11 mm^−1^
*T* = 296 K
0.80 × 0.22 × 0.21 mm

#### Data collection

Stoe IPDS-II diffractometer
Absorption correction: integration (*X-RED*; Stoe & Cie, 2002 ▶) *T*_min_ = 0.945, *T*_max_ = 0.982
8242 measured reflections
2501 independent reflections
1710 reflections with *I* > 2σ(*I*)
*R*_int_ = 0.033

#### Refinement

*R*[*F* ^2^ > 2σ(*F* ^2^)] = 0.038
*wR*(*F* ^2^) = 0.098
*S* = 1.02
2501 reflections
185 parameters
H atoms treated by a mixture of independent and constrained refinement
Δρ_max_ = 0.09 e Å^−3^
Δρ_min_ = −0.14 e Å^−3^

### 

Data collection: *X-AREA* (Stoe & Cie, 2002 ▶); cell refinement: *X-AREA*; data reduction: *X-RED32* (Stoe & Cie, 2002 ▶); program(s) used to solve structure: *SHELXS97* (Sheldrick, 2008 ▶); program(s) used to refine structure: *SHELXL97* (Sheldrick, 2008 ▶); molecular graphics: *ORTEP-3 for Windows* (Farrugia, 1997 ▶); software used to prepare material for publication: *WinGX* (Farrugia, 1999 ▶).

## Supplementary Material

Crystal structure: contains datablocks I, global. DOI: 10.1107/S1600536809008150/bt2883sup1.cif

Structure factors: contains datablocks I. DOI: 10.1107/S1600536809008150/bt2883Isup2.hkl

Additional supplementary materials:  crystallographic information; 3D view; checkCIF report

## Figures and Tables

**Table 1:** Hydrogen-bond geometry (Å, °)

*D*—H⋯*A*	*D*—H	H⋯*A*	*D*⋯*A*	*D*—H⋯*A*
O1—H1⋯N1	1.25 (3)	1.38 (3)	2.5547 (18)	153 (2)
C7—H7⋯O3^i^	0.93	2.46	3.3014 (19)	151
C10—H10⋯O1^ii^	0.93	2.57	3.4605 (18)	160

Symmetry codes: (i) 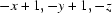; (ii) 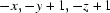.

**Fig. 1.**
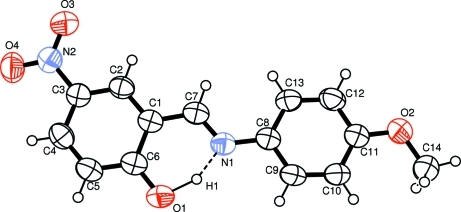
The molecular structure of the title compound showing the atom-numbering scheme and diplacement ellipsoids at the %50 probability.

**Fig. 2.**
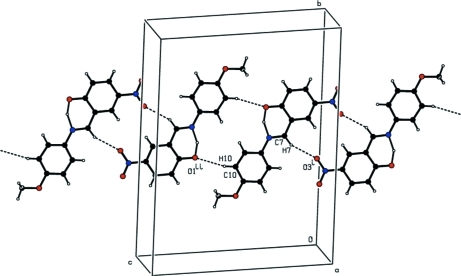
The crystal packing of the title compound. Intermolecular hydrogen bonds are shown as dashed lines.

## supplementary crystallographic information

### Comment

Schiff base compounds can be classified by their photochromic and thermochromic characteristics. Photochromism and thermochromism produced by the reversible intramolecular proton transfer associated with a change in π-electron configuration. Schiff bases display two possible tautomeric forms, the phenol-imine and the keto-amine forms. We report here on the crystal structure of the title compound, 2-[(4-Methoxyphenyl)iminomethyl]-4-nitrophenylen-1-olate, (I). The molecular structure of the compound (I) is described as an intermediate state between NH and OH tautomers. The bond lengths of the compound are intermediate between single and double C—O (1.362 and 1.222 Å, respectively) and C—N bond lengths (1.339 and 1.279 Å, respectively), (Allen *et al.*, 1987). In particular, C6—O1 bond length (1.318 Å) is significantly shorter than its expected value.

The molecular structure of 2-[(4-Methoxyphenyl)iminomethyl]-4- nitrophenylen-1-olate is shown in Fig. 1. The conformation is stabilized by an  intramolecular O—H···N hydrogen bond. It is a well known fact that H atoms participating in intramolecular hydrogen bonds in Schiff bases are rather mobile. The molecule can be regarded as having an intermediate state between its canonical OH and NH forms, and therefore the O1—H1 bond (1.246 Å) remains somewhat longer than its expected value.   On the other hand, the C3—N2 bond length [1.4521 (18) Å] in title compound is as expected and also is in agreement with the corresponding distances [1.4671 (18) Å (Zeller & Hunter, 2004) and 1.456 (4) Å (Glidewell *et al.*, 2004)] for compounds that contain a nitro group.

The molecule is nearly planar and the dihedral angle between the two benzene rings is 3.28 (7) Å. The crystal packing is stabilized by intermolecular C—H···O hydrogen bonds generating centrosymmetric *R*_2_^2^(18) and *R*_2_^2^(14) dimers.

### Experimental

The compound 2-[(4-Methoxyphenyl)iminomethyl]-4-nitrophenylen-1-olate was prepared by reflux a mixture of a solution containing 2-Hydroxy-5-nitrobenzaldehyde(0.0574 g 0.34 mmol) in 20 ml e thanol and a solution containing *p*-Anisidine (0.0423 g 0.34 mmol) in 20 ml e thanol. The reaction mixture was stirred for 1 hunder reflux. The crystals of (*E*)-2-[(4-Methoxyphenylimino)methyl]-4-nitrophenol suitable for X-ray analysis were obtained from ethylalcohol by slow evaporation (yield % 41; m.p.445–446 K).

### Refinement

All H atoms (expect for H1) were positioned geometrically and treated using a riding model, fixing the bond lengths at 0.93 and 0.96 Å for CH(aromatic) and CH_3_, respectively. The displacement parameters of the H atoms were constrained as *U*_iso_(H) = 1.2*U*_eq_(C_aromatic_) or 1.5*U*_eq_(C_methyl_). The position of the H1 atom was obtained from a difference map   and this atom was refined freely.

### Crystal data

**Table:**

C_14_H_12_N_2_O_4_	*F*(000) = 568
*M**_r_* = 272.26	*D*_x_ = 1.418 Mg m^−^^3^
Monoclinic, *P*2_1_/*c*	Mo *K*α radiation, λ = 0.71073 Å
Hall symbol: -P 2ybc	Cell parameters from 12745 reflections
*a* = 3.8883 (3) Å	θ = 1.6–28.9°
*b* = 21.6202 (17) Å	µ = 0.11 mm^−^^1^
*c* = 15.3127 (11) Å	*T* = 296 K
β = 97.887 (1)°	Prism, orange
*V* = 1275.10 (17) Å^3^	0.80 × 0.22 × 0.21 mm
*Z* = 4	

### Data collection

**Table:**

Stoe IPDS-II diffractometer	2501 independent reflections
Radiation source: fine-focus sealed tube	1710 reflections with *I* > 2σ(*I*)
plane graphite	*R*_int_ = 0.033
Detector resolution: 6.67 pixels mm^-1^	θ_max_ = 26.0°, θ_min_ = 1.6°
rotation method scans	*h* = −4→4
Absorption correction: integration (*X-RED*; Stoe & Cie, 2002)	*k* = −26→22
*T*_min_ = 0.945, *T*_max_ = 0.982	*l* = −18→18
8242 measured reflections	

### Refinement

**Table:**

Refinement on *F*^2^	Primary atom site location: structure-invariant direct methods
Least-squares matrix: full	Secondary atom site location: difference Fourier map
*R*[*F*^2^ > 2σ(*F*^2^)] = 0.038	Hydrogen site location: inferred from neighbouring sites
*wR*(*F*^2^) = 0.098	H atoms treated by a mixture of independent and constrained refinement
*S* = 1.02	*w* = 1/[σ^2^(*F*_o_^2^) + (0.0543*P*)^2^] where *P* = (*F*_o_^2^ + 2*F*_c_^2^)/3
2501 reflections	(Δ/σ)_max_ = 0.001
185 parameters	Δρ_max_ = 0.09 e Å^−^^3^
0 restraints	Δρ_min_ = −0.14 e Å^−^^3^

### Special details

**Table:**

Experimental. 168 frames, detector distance = 100 mm
Geometry. All e.s.d.'s (except the e.s.d. in the dihedral angle between two l.s. planes) are estimated using the full covariance matrix. The cell e.s.d.'s are taken into account individually in the estimation of e.s.d.'s in distances, angles and torsion angles; correlations between e.s.d.'s in cell parameters are only used when they are defined by crystal symmetry. An approximate (isotropic) treatment of cell e.s.d.'s is used for estimating e.s.d.'s involving l.s. planes.
Refinement. Refinement of *F*^2^ against ALL reflections. The weighted *R*-factor *wR* and goodness of fit *S* are based on *F*^2^, conventional *R*-factors *R* are based on *F*, with *F* set to zero for negative *F*^2^. The threshold expression of *F*^2^ > σ(*F*^2^) is used only for calculating *R*-factors(gt) *etc*. and is not relevant to the choice of reflections for refinement. *R*-factors based on *F*^2^ are statistically about twice as large as those based on *F*, and *R*- factors based on ALL data will be even larger.

### Fractional atomic coordinates and isotropic or equivalent isotropic displacement parameters (Å^2^)

**Table:**

	*x*	*y*	*z*	*U*_iso_*/*U*_eq_	
H1	0.080 (7)	0.5420 (15)	0.3247 (17)	0.159 (9)*	
C1	0.1593 (4)	0.54973 (7)	0.17787 (9)	0.0525 (4)	
C2	0.1968 (4)	0.55582 (7)	0.08919 (9)	0.0551 (4)	
H2	0.2959	0.5240	0.0603	0.066*	
C3	0.0878 (4)	0.60869 (7)	0.04447 (9)	0.0537 (4)	
C4	−0.0560 (4)	0.65778 (8)	0.08562 (10)	0.0615 (4)	
H4	−0.1280	0.6933	0.0541	0.074*	
C5	−0.0899 (4)	0.65300 (8)	0.17316 (10)	0.0639 (4)	
H5	−0.1815	0.6860	0.2014	0.077*	
C6	0.0109 (4)	0.59926 (7)	0.22101 (9)	0.0566 (4)	
C7	0.2677 (4)	0.49350 (7)	0.22438 (9)	0.0569 (4)	
H7	0.3680	0.4620	0.1951	0.068*	
C8	0.3240 (4)	0.43228 (7)	0.35558 (9)	0.0532 (4)	
C9	0.2812 (4)	0.43386 (7)	0.44334 (9)	0.0591 (4)	
H9	0.1954	0.4697	0.4661	0.071*	
C10	0.3611 (4)	0.38411 (8)	0.49849 (9)	0.0594 (4)	
H10	0.3300	0.3863	0.5576	0.071*	
C11	0.4880 (4)	0.33088 (7)	0.46477 (9)	0.0554 (4)	
C12	0.5339 (4)	0.32849 (8)	0.37667 (10)	0.0654 (4)	
H12	0.6206	0.2927	0.3541	0.078*	
C13	0.4526 (4)	0.37842 (8)	0.32228 (9)	0.0627 (4)	
H13	0.4836	0.3762	0.2632	0.075*	
C14	0.5188 (5)	0.27857 (8)	0.60288 (10)	0.0719 (5)	
H14A	0.5916	0.2397	0.6295	0.108*	
H14B	0.2760	0.2846	0.6059	0.108*	
H14C	0.6495	0.3114	0.6339	0.108*	
N1	0.2277 (3)	0.48627 (6)	0.30559 (7)	0.0567 (3)	
N2	0.1205 (4)	0.61313 (7)	−0.04865 (8)	0.0617 (3)	
O1	−0.0338 (3)	0.59470 (6)	0.30459 (7)	0.0713 (3)	
O2	0.5770 (3)	0.27862 (5)	0.51285 (7)	0.0696 (3)	
O3	0.2895 (3)	0.57320 (6)	−0.08103 (7)	0.0806 (4)	
O4	−0.0247 (4)	0.65529 (6)	−0.09199 (7)	0.0860 (4)	

### Atomic displacement parameters (Å^2^)

**Table:**

	*U*^11^	*U*^22^	*U*^33^	*U*^12^	*U*^13^	*U*^23^
C1	0.0452 (8)	0.0534 (9)	0.0586 (8)	−0.0031 (7)	0.0055 (6)	−0.0084 (6)
C2	0.0523 (9)	0.0528 (9)	0.0607 (8)	−0.0028 (7)	0.0094 (7)	−0.0089 (7)
C3	0.0515 (9)	0.0527 (9)	0.0560 (8)	−0.0061 (7)	0.0040 (6)	−0.0060 (6)
C4	0.0566 (9)	0.0532 (9)	0.0721 (10)	0.0006 (8)	−0.0001 (7)	−0.0049 (7)
C5	0.0640 (10)	0.0580 (10)	0.0692 (9)	0.0066 (8)	0.0079 (7)	−0.0129 (7)
C6	0.0483 (9)	0.0590 (10)	0.0619 (9)	−0.0026 (7)	0.0051 (6)	−0.0121 (7)
C7	0.0521 (9)	0.0580 (10)	0.0610 (9)	−0.0009 (7)	0.0090 (6)	−0.0106 (7)
C8	0.0478 (8)	0.0545 (9)	0.0572 (8)	−0.0005 (7)	0.0069 (6)	−0.0073 (6)
C9	0.0586 (9)	0.0595 (10)	0.0604 (9)	0.0067 (8)	0.0127 (7)	−0.0117 (7)
C10	0.0589 (9)	0.0651 (10)	0.0553 (8)	0.0024 (8)	0.0114 (7)	−0.0094 (7)
C11	0.0476 (8)	0.0576 (10)	0.0605 (8)	−0.0018 (7)	0.0055 (6)	−0.0051 (7)
C12	0.0732 (11)	0.0570 (10)	0.0671 (9)	0.0064 (8)	0.0132 (7)	−0.0126 (7)
C13	0.0708 (11)	0.0648 (10)	0.0538 (8)	0.0036 (8)	0.0129 (7)	−0.0099 (7)
C14	0.0777 (12)	0.0709 (11)	0.0676 (10)	0.0007 (10)	0.0113 (8)	0.0033 (8)
N1	0.0550 (8)	0.0589 (8)	0.0562 (7)	−0.0001 (6)	0.0078 (5)	−0.0069 (5)
N2	0.0646 (8)	0.0566 (8)	0.0632 (8)	−0.0096 (7)	0.0058 (6)	−0.0036 (6)
O1	0.0835 (9)	0.0729 (8)	0.0594 (6)	0.0080 (6)	0.0166 (5)	−0.0120 (5)
O2	0.0800 (8)	0.0604 (7)	0.0687 (7)	0.0070 (6)	0.0115 (5)	−0.0007 (5)
O3	0.0992 (10)	0.0787 (9)	0.0677 (7)	0.0089 (7)	0.0251 (6)	−0.0054 (6)
O4	0.1139 (11)	0.0697 (8)	0.0719 (7)	0.0082 (8)	0.0038 (6)	0.0113 (6)

### Geometric parameters (Å, °)

**Table:**

C1—C2	1.3917 (19)	C9—C10	1.376 (2)
C1—C6	1.421 (2)	C9—H9	0.9300
C1—C7	1.442 (2)	C10—C11	1.380 (2)
C2—C3	1.370 (2)	C10—H10	0.9300
C2—H2	0.9300	C11—O2	1.3669 (18)
C3—C4	1.390 (2)	C11—C12	1.386 (2)
C3—N2	1.4521 (18)	C12—C13	1.374 (2)
C4—C5	1.369 (2)	C12—H12	0.9300
C4—H4	0.9300	C13—H13	0.9300
C5—C6	1.401 (2)	C14—O2	1.4275 (17)
C5—H5	0.9300	C14—H14A	0.9600
C6—O1	1.3186 (16)	C14—H14B	0.9600
C7—N1	1.2837 (17)	C14—H14C	0.9600
C7—H7	0.9300	N1—H1	1.38 (3)
C8—C9	1.3770 (19)	N2—O4	1.2196 (17)
C8—C13	1.391 (2)	N2—O3	1.2297 (16)
C8—N1	1.4177 (19)	O1—H1	1.25 (3)
			
C2—C1—C6	119.07 (14)	C9—C10—C11	118.97 (14)
C2—C1—C7	119.93 (13)	C9—C10—H10	120.5
C6—C1—C7	121.01 (13)	C11—C10—H10	120.5
C3—C2—C1	120.01 (14)	O2—C11—C10	124.40 (13)
C3—C2—H2	120.0	O2—C11—C12	115.88 (14)
C1—C2—H2	120.0	C10—C11—C12	119.71 (15)
C2—C3—C4	121.78 (14)	C13—C12—C11	120.75 (15)
C2—C3—N2	118.91 (13)	C13—C12—H12	119.6
C4—C3—N2	119.31 (14)	C11—C12—H12	119.6
C5—C4—C3	119.01 (15)	C12—C13—C8	119.99 (14)
C5—C4—H4	120.5	C12—C13—H13	120.0
C3—C4—H4	120.5	C8—C13—H13	120.0
C4—C5—C6	121.16 (15)	O2—C14—H14A	109.5
C4—C5—H5	119.4	O2—C14—H14B	109.5
C6—C5—H5	119.4	H14A—C14—H14B	109.5
O1—C6—C5	120.34 (14)	O2—C14—H14C	109.5
O1—C6—C1	120.71 (14)	H14A—C14—H14C	109.5
C5—C6—C1	118.95 (13)	H14B—C14—H14C	109.5
N1—C7—C1	121.00 (14)	C7—N1—C8	124.42 (13)
N1—C7—H7	119.5	C7—N1—H1	102.0 (11)
C1—C7—H7	119.5	C8—N1—H1	133.6 (11)
C9—C8—C13	118.42 (15)	O4—N2—O3	122.58 (14)
C9—C8—N1	116.64 (13)	O4—N2—C3	119.16 (14)
C13—C8—N1	124.94 (13)	O3—N2—C3	118.24 (14)
C10—C9—C8	122.15 (15)	C6—O1—H1	102.6 (12)
C10—C9—H9	118.9	C11—O2—C14	117.30 (13)
C8—C9—H9	118.9		
			
C6—C1—C2—C3	−0.7 (2)	C8—C9—C10—C11	−0.1 (2)
C7—C1—C2—C3	178.72 (14)	C9—C10—C11—O2	179.88 (14)
C1—C2—C3—C4	1.2 (2)	C9—C10—C11—C12	0.3 (2)
C1—C2—C3—N2	−178.21 (12)	O2—C11—C12—C13	179.99 (15)
C2—C3—C4—C5	−0.1 (2)	C10—C11—C12—C13	−0.4 (2)
N2—C3—C4—C5	179.23 (13)	C11—C12—C13—C8	0.3 (2)
C3—C4—C5—C6	−1.3 (2)	C9—C8—C13—C12	−0.1 (2)
C4—C5—C6—O1	−177.85 (14)	N1—C8—C13—C12	−179.22 (15)
C4—C5—C6—C1	1.7 (2)	C1—C7—N1—C8	179.33 (13)
C2—C1—C6—O1	178.89 (13)	C9—C8—N1—C7	176.31 (14)
C7—C1—C6—O1	−0.6 (2)	C13—C8—N1—C7	−4.5 (2)
C2—C1—C6—C5	−0.7 (2)	C2—C3—N2—O4	168.40 (14)
C7—C1—C6—C5	179.89 (14)	C4—C3—N2—O4	−11.0 (2)
C2—C1—C7—N1	−178.28 (13)	C2—C3—N2—O3	−10.2 (2)
C6—C1—C7—N1	1.2 (2)	C4—C3—N2—O3	170.37 (14)
C13—C8—C9—C10	0.0 (2)	C10—C11—O2—C14	3.0 (2)
N1—C8—C9—C10	179.19 (14)	C12—C11—O2—C14	−177.33 (14)

### Hydrogen-bond geometry (Å, °)

**Table:**

*D*—H···*A*	*D*—H	H···*A*	*D*···*A*	*D*—H···*A*
O1—H1···N1	1.25 (3)	1.38 (3)	2.5547 (18)	153 (2)
C7—H7···O3^i^	0.93	2.46	3.3014 (19)	151
C10—H10···O1^ii^	0.93	2.57	3.4605 (18)	160

Symmetry codes: (i) −*x*+1, −*y*+1, −*z*; (ii) −*x*, −*y*+1, −*z*+1.
